# High Sensitivity Protein Gel Electrophoresis Label Compatible with Mass-Spectrometry

**DOI:** 10.3390/bios10110160

**Published:** 2020-10-31

**Authors:** Joshua A. Welsh, Lisa M. Jenkins, Julia Kepley, Gaelyn C. Lyons, David M. Moore, Tim Traynor, Jay A. Berzofsky, Jennifer C. Jones

**Affiliations:** 1 Laboratory of Pathology, Centre for Cancer Research, National Cancer Institute, National Institutes of Health, Bethesda, MD 20892, USA; joshua.welsh@nih.gov (J.A.W.); jak332@georgetown.edu (J.K.); dmitchellmoore@hotmail.com (D.M.M.); tim.traynor@nih.gov (T.T.); 2 Laboratory of Cell Biology, Centre for Cancer Research, National Cancer Institute, National Institutes of Health, Bethesda, MD 20892, USA; lisa.jenkins@nih.gov (L.M.J.); glyons1@pennstatehealth.psu.edu (G.C.L.); 3 Vaccine Branch, Centre for Cancer Research, National Cancer Institute, National Institutes of Health, Bethesda, MD 20892, USA; berzofsj@mail.nih.gov

**Keywords:** extracellular vesicles, gel electrophoreses, labelling, mass spectrometry, protein

## Abstract

Sodium dodecyl sulfate polyacrylamide gel electrophoresis (SDS-PAGE) is a widely utilized technique for macromolecule and protein analysis. While multiple methods exist to visualize the separated protein bands on gels, one of most popular methods of staining the proteins is with Coomassie dye. A more recent approach is to use Bio-Rad stain-free technology for visualizing protein bands with UV light and achieve similar or greater sensitivity than that of Coomassie dye. Here, we developed a method to further amplify the sensitivity of stain-free gels using carboxyfluorescein succinimidyl ester (CFSE) staining. We compared our novel method using foetal bovine serum samples with Coomassie dye, standard stain-free gels, and silver staining. Our results show that while silver staining remains a gold-standard method in terms of sensitivity; CFSE staining of samples prior to use with stain-free gels results in a 10–100-fold increase in sensitivity over Coomassie staining and the standard stain-free method. Our method offers a sensitivity similar to that of silver staining which is compatible with downstream mass spectrometry, and therefore more advantageous for further retrieval and analysis of macromolecules in bands.

## 1. Introduction

Sodium dodecyl sulfate polyacrylamide gel electrophoresis (SDS-PAGE) is a widely utilized technique for analyzing macromolecules such as proteins and nucleic acids [1,2,3]. In order to identify the separation and size of macromolecules, a number of labeling compounds are commonly used including Coomassie dye, zinc, fluorescent dye, and silver stain [3,4,5]. Each staining method has its advantages and disadvantages. In terms of total protein detection, for example, silver staining is considered to be the most sensitive technique. However, the staining protocol is time-consuming and results are easily affected by a number of factors such as reagent quality, incubation times, and gel thickness [3]. Another drawback of many of these stains is that many are not compatible with downstream analysis methods such as Western blotting and mass spectrometry [6].

Recently, a new technology has gained popularity in SDS-PAGE analysis, i.e., stain-free gels. Stain-free gels are composed, in part, of trihalocompounds which covalently bind to tryptophan, an amino acid found in most protein samples, to fluoresce under ultraviolent (UV) light [3,6,7]. Thus, stain-free gels do not require a post-electrophoretic staining step; rather, they can be activated and analyzed under UV light following electrophoresis. Studies have shown that these gels have the ability to detect protein samples as small as 20–50 ng, which is a lower detection limit than that of some Coomassie blue stains [7]. In addition to exhibiting high sensitivity and a low detection limit, stain-free gels are compatible with many downstream applications [6]. The reported sensitivity of protein staining methods, however, varies greatly, with manufacturers claiming sensitivity of Coomassie staining methods as low as 3–10 ng, silver staining methods 0.25–5 ng, fluorescent staining 0.25–8 ng, and stain-free methods 0.25–5 ng [8,9].

While stain-free technology is designed to work without additional protein staining, it is possible to prelabel protein samples with various fluorophores to increase their signal intensity. One such compound of interest is carboxyfluorescein diacetate succinimidyl ester (CFDA-SE). While technically classified as a non-fluorescent compound [10], CFDA-SE is easily converted into a fluorescent compound (carboxyfluorescein succinimidyl ester (CFSE)) through hydrolytic degradation which occurs when CFSE binds to amino groups on proteins, releasing the succinimide moiety. CFDA-SE is commonly used to monitor the frequency of cell division due to its ability to diffuse through the plasma membrane and stably bind to intracellular proteins [10,11]. When a cell divides, each daughter cell obtains half of the original CFSE, enabling researchers to visualize this process. CFSE exhibits a relative excitation intensity of around 50% at UV wavelengths (emission peak at 517 nm) [9]. This property makes it a promising compound for enhancing protein detection in stain-free gels, which are also activated by UV light. Here, we demonstrate a method of combining CFSE labeling with stain-free gel technology to enhance the sensitivity of detection for protein samples.

## 2. Materials and Methods

### 2.1. Carboxyfluorescein Succinimidyl Ester (CFSE) Labeling

**Figure 1:**
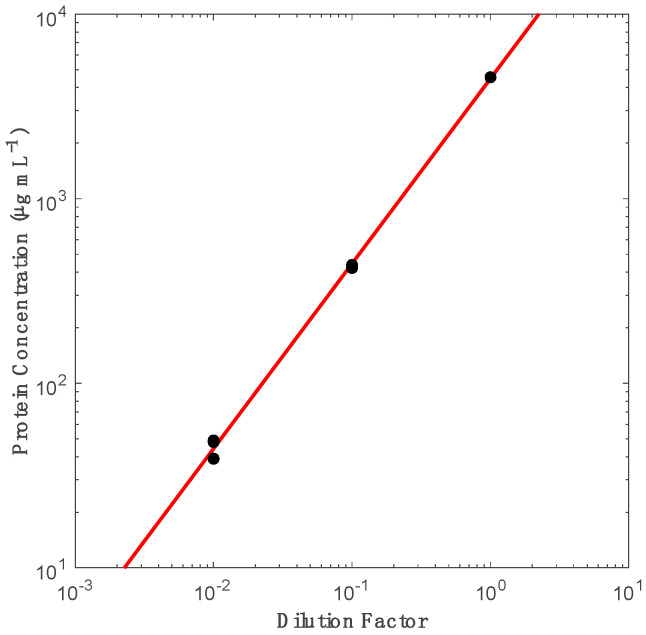
NanoDrop protein concentration approximation using serially diluted fetal bovine serum (FBS). Regression was performed with data from serially diluted FBS, with three measurements shown per dilution.

Fetal bovine serum (FBS) (Thermo Fisher Scientific, Waltham, MA, USA, 4557.7 µg mL^−1^ concentration) was analyzed to confirm protein concentration using NanoDrop 2000 (Thermo Fisher Scientific), and then used to make five 250 µL decreasing dilutions (stock, 10×, 100×, 1000×, 10,000×) in DPBS (Thermo Fisher Scientific). The theoretical concentrations of the lowest two dilutions were approximated based on extrapolating from the trendline established by the first three data points that were within the detection limits of the instruments, Figure 1. Then, these corrected concentration values were used to calculate the approximate amount of protein (ng) in each band. Protein quantification using NanoDrop is based on Beer–Lamber Law (A=εbC), where the absorbance (A) reading, can derive the concentration (C) by having a known path length (b) and molar absorptivity (ε). The default setting being that 1 absorbance units is equal to 1 mg mL^−1^ protein. While there are limitations to the absolute protein quantification, NanoDrop was selected for its ergonomics and high dynamic range as compared with more sensitive methods. Previous work has indicated that variations in protein determination from complex fluids could vary between protein quantification methods, with NanoDrop capable of estimating protein concentration within 30% of more sensitive methods such as µBCA assays, Supplementary Figure S1. Then, 100 µL of each dilution were mixed with 16 µL of 0.1 mM CFSE (Thermo Fisher Scientific) in individual wells of a Crystalgen RNAase/DNAse free 12-PCR tube strip. Five additional wells were filled with 100 µL of the same FBS dilutions without the CFSE stain. Then, the PCR strip was vortexed, spun down, and covered in aluminum foil to incubate in an Eppendorf Thermomixer for 2 h, at 37 °C. After incubation, 25 µL of each FBS sample, both CFSE stained and unstained, were mixed with 1.25 µL 2-mercaptoethanol (Thermo Fisher Scientific) and 13.75 µL Laemmli sample buffer (Bio-Rad), and denatured at 95 °C for 10 min using a GeneAmp PCR System 9700.

### 2.2. SDS-PAGE

A 10% Tris/Glycine/SDS Buffer solution was prepared with 100 mL buffer (Bio-Rad)/900 mL tissue culture grade water (TCW). A custom ladder was created with a mixture of 35 µL unstained and 35 µL all-blue ladders, and then 10 µL of the custom ladder were added to the first and the last wells of three Bio-Rad Mini-PROTEAN TGX gels, two unstained and one stain-free gel. All ten FBS samples (5 dilutions with CFSE, 5 dilutions without CFSE) were pipetted into each of the three gels in 10 µL aliquots. With a Mini-PROTEAN Tetra System connected to a Bio-Rad Power Pac 1000, SDS-PAGE was run at a constant voltage of 200 V until the bands ran off the bottom of the gels. While the unstained gels were, then, prepped using two traditional gel stains, Coomassie blue and silver stain as described below, the stain-free gel was imaged under the Bio-Rad ChemiDoc Touch Imaging System.

### 2.3. Coomassie Blue Stain Preparation

One of the unstained gels was thoroughly rinsed in TCW and left to soak in 20 mL of GelCode Blue Safe Protein Stain (Thermo Fisher Scientific). After 15 min, the stain was removed and replaced with 20 mL TCW. Every 10 min for the next hour and a half, the rinse water was decanted and exchanged with fresh TCW to fully remove any excess blue stain. The gel was then analyzed under the Bio-Rad imaging system with the Coomassie blue setting.

### 2.4. Silver Stain Preparation

To create the stain, the following five different solutions were prepared using a SilverXpress Silver Staining Kit: a fixing solution (90 mL TCW, 100 mL methanol, 20 mL acetic acid), a sensitizing solution (105 mL TCW, 100 mL methanol, 5 mL sensitizer), a staining solution (5 mL stainer A, 5 mL stainer B, 90 mL TCW), a developing solution (95 mL TCW, 5 mL developer), and a stopping solution (5 mL stopper). The second unstained gel was initially placed in 200 mL of the fixing solution for 10 min. After decanting the solution, the gel was incubated in 100 mL of the sensitizing solution for another 10 min. This process was repeated twice before decanting the second sensitizing solution and soaking it for two 5 min intervals with 200 mL TCW. Then, the gel was incubated in 100 mL staining solution for 15 min and rinsed twice with 200 mL TCW. After applying the stain, the gel was soaked in 100 mL developing solution. Once an optimal staining intensity was reached, 5 mL of stopping solution were added, and then decanted. Finally, the gel was washed thoroughly in 200 mL of TCW before analysis with the silver stain setting under the Bio-Rad imaging system.

### 2.5. Mass Spectrometry

FBS samples, either CFSE stained or unstained, were separated by SDS-PAGE and each lane cut into 10 slices. Then, the protein bands were in-gel digested with trypsin (Thermo Fisher Scientific) overnight, at 37 °C, as described [12]. The peptides were extracted following cleavage and lyophilized. The dried peptides were solubilized in 2% acetonitrile, 0.5% acetic acid, 97.5% water for mass spectrometry analysis; percentages refer to volume per volume. They were trapped on a trapping column and separated on a 75 µm × 15 cm, 2 µm Acclaim PepMap reverse phase column (Thermo Fisher Scientific) using an UltiMate 3000 RSLCnano HPLC (Thermo Fisher Scientific). Peptides were separated at a flow rate of 300 nL min^−1^ followed by online analysis by tandem mass spectrometry using a Thermo Orbitrap Fusion mass spectrometer. Peptides were eluted into the mass spectrometer using a linear gradient from 96% mobile phase A (0.1% formic acid in water) to 55% mobile phase B (0.1% formic acid in acetonitrile) over 30 min. Parent full scan mass spectra were collected in the Orbitrap mass analyzer set to acquire data at 120,000 FWHM resolution; then, ions were isolated in the quadrupole mass filter, fragmented within the HCD cell (HCD normalized energy 32%, stepped ± 3%), and the product ions analyzed in the ion trap. Proteome Discoverer 2.2 (Thermo Fisher Scientific) was used to search the data against bovine proteins from the UniProt database using SequestHT. The search was limited to tryptic peptides, with maximally two missed cleavages allowed. Cysteine carbamidomethylation was set as a fixed modification, and methionine oxidation set as a variable modification. The precursor mass tolerance was 10 ppm, and the fragment mass tolerance was 0.6 Da. The Percolator node was used to score and rank peptide matches using a 1% false discovery rate. In analyzing the mass spectrometry tryptic peptide data, we did not include potential modification by CFSE to allow for direct comparison between the two samples and to match standard sample processing methods.

## 3. Results

### 3.1. Effect of Protein Reduction Methods on CFSE

**Figure 2:**
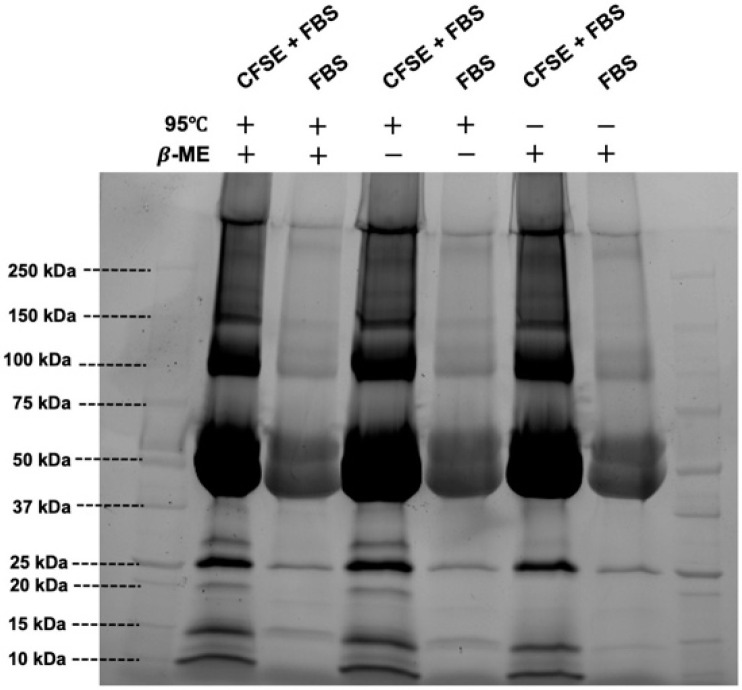
The effect of standard reduction methods upon carboxyfluorescein succinimidyl ester (CFSE)-labeled samples. Standard reduction methods include 95 °C for 10 min and 2-mercaptoethanol used alone and in combination with CFSE-stained and unstained samples on an SDS-PAGE stain-free gel.

The two commonly used methods, incubation in a high temperature (95 °C) and incubation with the denaturing agent 2-mercaptoethanol (β-ME) to cleave disulfide bonds, were investigated. First, 5 µL of CFSE-stained fetal bovine serum (FBS) were diluted in 1 mL Dulbecco’s phosphate buffered saline (DPBS), and then subjected to either 95 °C, treatment with β-ME, or both. Then, 25 µL of each group were pipetted into each well and their resulting fluorescence intensities were compared on the gel. As displayed in Figure 2, the bands corresponding to the +/+, +/−, and −/+ (high temperature/β-ME treatment) samples were qualitatively identical in terms of fluorescence intensity.

### 3.2. Influence of CFSE Staining on FBS Detectability

**Figure 3:**
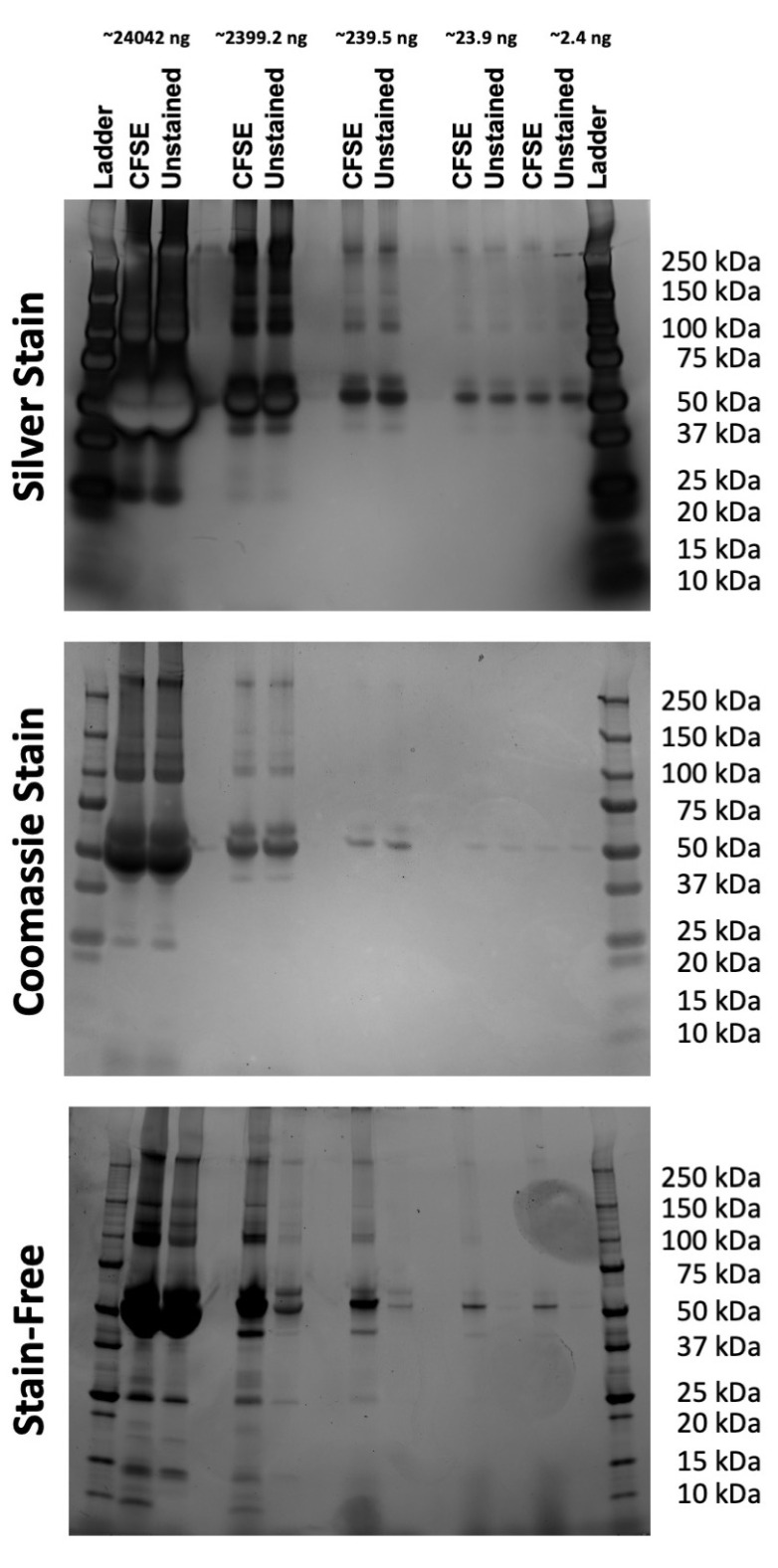
Sensitivity comparison of silver stain, Coomassie blue, and stain-free methodologies with the same loaded FBS samples with and without CFSE staining.

Unstained FBS and CFSE-stained FBS samples ranging five orders of magnitude in protein quantity were run on gels with silver staining, Coomassie staining, and stain-free methodologies, Figure 3. CFSE staining did not influence the detectability using silver-staining or Coomassie staining methods. CFSE staining did, however, increase the detectability of proteins using the stain-free methodology by ~100-fold. While the unstained FBS protein bands became nondetectable at a total protein input amount between ~239.5 and ~23.9 ng, the CFSE-stained FBS remained detectable at an amount of ~2.4 ng. The sensitivity of the stain-free gel alone was fairly comparable if not slightly higher than that of the Coomassie stained gel. However, both the Coomassie gel and unlabeled stain-free gel had far higher thresholds for detection than both the CFSE-labeled stain-free gel and the silver stained gel. The latter two gels allowed for the visualization of all five FBS amounts, demonstrating their usefulness in separating and analyzing extremely minimal protein amounts.

### 3.3. Influence of CFSE Staining on Downstream Mass Spectrometry

**Figure 4:**
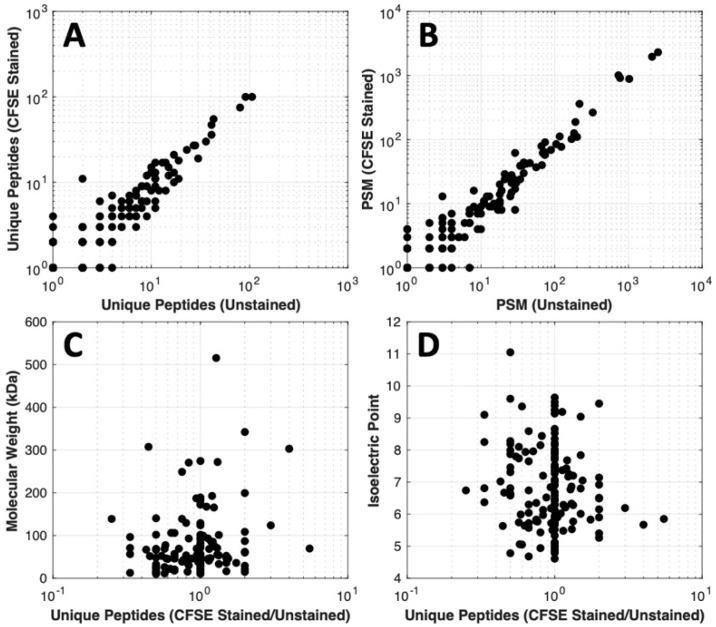
Mass spectrometry data comparison of CFSE stained and unstained samples. Proteins were in-gel trypsin digested and analyzed by tandem mass spectrometry. Scatter diagrams. (**A**) The number of unique peptides detected per protein from stained vs. unstained samples; (**B**) Ratio of unique peptides in the CFSE stained over unstained vs. molecular weight; (**C**) Number of peptide spectra matches per protein in the stained vs. unstained samples; (**D**) Ratio of unique peptides in the CFSE stained over unstained vs. isoelectric point.

Mass spectrometry of unstained FBS and CFSE-stained FBS was carried out to determine if CFSE impaired downstream assays, Figure 4. When comparing the proteins identified by the mass spectrometry analysis, the resulting data was linear with no outliers or bias toward unstained or stained samples. This supports that the CFSE staining did not cause any noticeable change in the number of unique peptide sequences detected per protein or number of peptide spectral matches per protein, indicating that the proteins identified were similarly identified without and with CFSE treatment, Figure 4A,B. Furthermore, the isoelectric points and molecular weights of the proteins identified were similar and did not show a clear bias toward CFSE stained or unstained samples, suggesting that there was no preferential effect of the CFSE stain on proteins of a particular size or charge Figure 4C,D. Thus, the CFSE stain does not substantially modify any particular molecule of protein in such a way that would impair downstream mass spectrometry analysis; moreover, these results demonstrate that there is not a preferential modification of a size or charge of protein by CFSE, such that all proteins are likely modified to a similar, low extent.

## 4. Discussion

In this research, we attempted to enhance the sensitivity and detection range of stain-free electrophoresis gels by prelabeling FBS protein samples with the fluorescent compound CFSE. Since CFSE is compatible with stain-free gel technology, it requires no post-electrophoretic manipulation, and therefore is more ergonomic than traditional Coomassie-staining procedures. First, we demonstrated that proteins labeled with CFSE could be denatured using standard protein fixation protocols. This data demonstrates that the proposed CFSE staining technique does not require a different pre-electrophoretic preparation than current staining methods. Secondly, we demonstrated that CFSE-labeled FBS proteins were still visible at a 100-fold lower protein input as compared with Coomassie and stain-free gels alone. Silver staining did, however, have the highest detectability of low abundant proteins. Finally, we demonstrated that CFSE labeling was fully compatible with downstream mass spectrometry analysis without requiring a method of destaining, while conventional silver staining was not [13]. As prior literature suggests, the silver staining provides the highest sensitivity out of the compared stains [14]. Nevertheless, CFSE still exhibited excellent sensitivity and could be used as an easier alternative to silver stain to visualize most protein samples. Further research would need to be conducted to determine the absolute detection limit and residue staining bias of the silver stain as comparedi with the CFSE-labeled stain-free gel.

In summary, the high sensitivity, relatively easy preparation, and compatibility with other downstream analysis techniques warrants the consideration of CFSE staining of samples in combination with stain-free gel technology as a novel attractive protein stain amplification for SDS-PAGE applications. This method of detection may be useful for applications within the extracellular vesicle field, in which concentrating the vesicles to a detectable level with conventional methods is hard to achieve with small sample sizes.

## Supplementary Materials

The following are available online at https://www.mdpi.com/2079-6374/10/11/160/s1, Figure S1: A standard curve (left plot) was created using protein standards included with the µBCA assay (Thermo Fisher Scientific). The standard curve was used to derive the protein concentration of 4 different isolations of extracellular vesicle-rich tissue culture medium. These samples were also measured using NanoDrop with datapoints for each technique plotted against one another (right plot). NanoDrop consistently overestimated protein concentration by 13 to 29%.

## References

[1] Carter J., Petersen B., Printz S., Sorey T., Kroll T. (2013). Quantitative Application for SDS-PAGE in a Biochemistry Lab. J. Chem. Educ..

[2] Svasti J., Panijpan B. (1977). SDS-Polyacrylamide Gel Electrophoresis. J. Chem. Educ..

[3] Yadav G., Liu N. (2014). Trends in Protein Separation and Analysis- the Advance of Stain-Free Technology. BioRadiations.

[4] Panfoli I., Calzia D., Santucci L., Ravera S., Bruschi M., Candiano G. (2012). A blue dive: From ‘blue fingers’ to ‘blue silver’. A comparative overview of staining methods for in-gel proteomics. Expert Rev. Proteom..

[5] Chiari M., Nesi M., Roncada P., Righetti P.G. (1994). Preparative isoelectric focusing in multicompartment electrolyzers: Novel, hydrolytically stable and hydrophilic isoelectric membranes. Electrophoresis.

[6] Elbaggari A., Choe J., McDonald K., Alburo A. (2008). Evaluation of the Criterion Stain-Free Gel Imaging System for Use in Western Blotting Applications. Imaging.

[7] Ladner C.L., Edwards R.A., Schreimer D.C., Turner R.J. (2006). Identification of Trichloroethanol Visualized Proteins from Two-Dimensional Polyacrylamide Gels by Mass Spectrometry. Anal. Chem..

[8] Bio-Rad https://www.bioradiations.com/trends-in-protein-separation-and-analysis-the-advance-of-stain-free-technology/.

[9] Thermo Fisher Fluorescence Spectraviewer. https://www.ThermoFisher.com/us/en/home/life-science/cell-analysis/labeling-chemistry/fluorescence-spectraviewer.html.

[10] Azari H., Deleyrolle L.P., Reynolds B.A. (2018). Using Carboxy Fluorescein Succinimidyl Ester (CFSE) to Identify Quiescent Glioblastoma Stem-Like Cells.

[11] Lyons A.B. (2000). Analysing cell division in vivo and in vitro using flow cytometric measurement of CFSE dye dilution. J. Immunol. Methods.

[12] Shevchenko A., Tomas H., Havlis J., Olsen J.V., Mann M. (2006). In-gel digestion for mass spectrometric characterization of proteins and proteomes. Nat. Protoc..

[13] Stochaj W.R., Berkelman T., Laird N. (2007). Mass spectrometry-compatible silver staining. CSH Protoc..

[14] Winkler C., Denker K., Wortelkamp S., Sickmann A. (2007). Silver- and Coomassie-staining protocols: Detection limits and compatibility with ESI MS. Electrophoresis.

