# Supplementary material for: High Sensitivity Protein Gel Electrophoresis Label Compatible with Mass-Spectrometry
Source: Biosensors (Basel). 2020 Oct 31;10(11):160. doi: 10.3390/bios10110160 (PMC7694097; doi:10.3390/bios10110160)
Supplement: Supplementary file 1 [file biosensors-10-00160-s001.pdf]

biosensors

MDPI

Supplementary materials

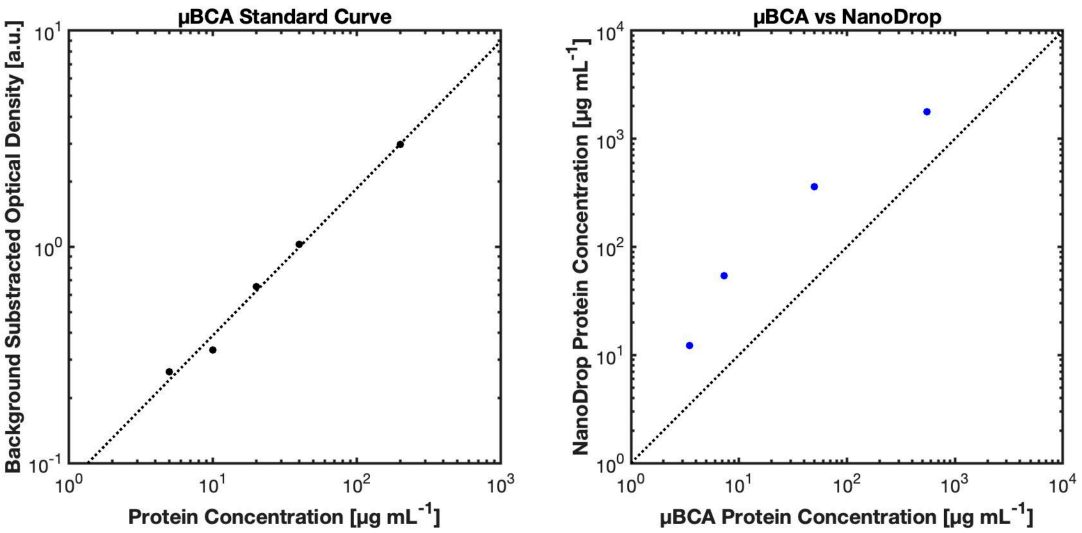

Supplementary Figure S1. A standard curve (left plot) was created using protein standards included with the µBCA assay (ThermoFisher Scientific). The standard curve was used to derived the protein concentration of 4 different isolations of extracellular vesicle-rich tissue culture medium. These samples were also measured using NanoDrop with datapoints for each technique plotted against one another (right plot). NanoDrop consistently overestimated protein concentration by 13 to 29 %.
